# *Mycobacterium arupense* as an Emerging Cause of Tenosynovitis

**DOI:** 10.3201/eid2203.151479

**Published:** 2016-03

**Authors:** Fiorella Krapp Lopez, Madeline Miley, Babafemi Taiwo

**Affiliations:** Northwestern University, Chicago, Illinois, USA (F. Krapp Lopez, B. Taiwo);; Northwestern Memorial Hospital, Chicago (M. Miley)

**Keywords:** *Mycobacterium arupense*, nontuberculous *Mycobacterium* infections, tenosynovitis, canakinumab, corticosteroids, tuberculosis and other mycobacteria, bacteria

**To the Editor:** *Mycobacterium arupense* was identified in 2006 as a novel species within the *M. terrae* complex with close similarity to *M. nonchromogenicum* (*1*). Since then, 8 cases describing clinically notable disease have been published (*2*–*8*), including 5 cases of tenosynovitis. We report *M. arupense* tenosynovitis in an immunocompromised person who received the selective interleukin (IL) 1 β-inhibitor canakinumab.

In July 2014, a 62-year-old man sought treatment at the emergency department, Northwestern Memorial Hospital (Chicago, Illinois, USA), after 1 week of pain and swelling in the right hand. During the previous 5 years, he had received multiple immunomodulatory drugs for treatment of natural killer cell deficiency, hyper–IL-6 syndrome, recurrent polychondritis, and Sweet syndrome. His medications were prednisone (42.5 mg/d), intravenous immunoglobulin (400 mg/kg monthly), and subcutaneous canakinumab (180 mg every 8 weeks, which began 3 weeks before onset of symptoms).

His first symptom was a tender red nodule on the right palm that increased in size and became extremely tender over the following week (Figure, panels A, B). He did not recall any trauma and denied fever or chills. No improvement was seen after he received oral linezolid for 5 days. A skin punch biopsy specimen showed a neutrophilic interstitial infiltrate with no granulomas; results of microbiological stains, including acid-fast bacilli, were negative, . His prednisone dosage was increased to 60 mg/d for suspected Sweet syndrome and, subsequently, to 80 mg/d when no improvement was observed after 2 weeks. A second dose of canakinumab was administered 8 weeks after the first. Shortly after, he was readmitted to the hospital with progression of edema and pain and signs consistent with carpal tunnel syndrome and trigger finger syndrome of the right index finger. Magnetic resonance imaging showed extensive tenosynovitis of the carpal tunnel flexor tendons and no bone erosions. Surgical release and tenosynovectomy of the carpal tunnel was performed; pathologic features demonstrated chronic inflammation of the synovium and absence of granulomas. Results of microbiological stains were negative.

**Figure:**
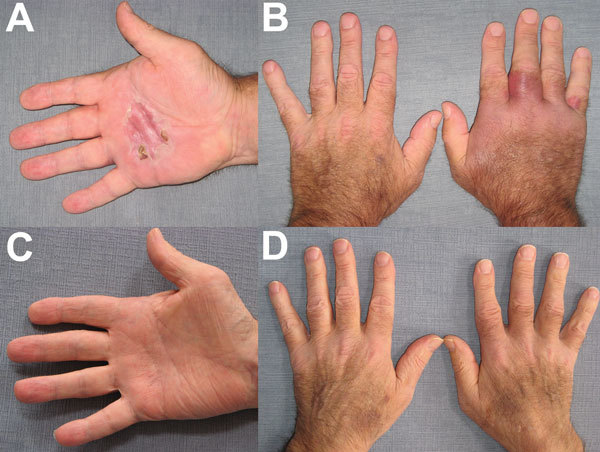
Hands of a 62-year-old man in Chicago, Illinois, USA, who had *Mycobacterium arupense* tenosynovitis, at the time treatment was sought (panels A, B) and after 6 months of treatment (panels C, D).

*M. arupense* grew on Löwenstein-Jensen culture from the skin biopsy specimen after 35 days and from a synovium specimen after 22 days. No growth was observed on liquid culture media. Empiric treatment was started immediately after the first positive culture: clarithromycin (500 mg 2×/d), ethambutol (1,200 mg/d), and rifabutin (300 mg/d). Prednisone was decreased to 45 mg/d, and canakinumab was discontinued. Susceptibility testing confirmed the *M. arupense* strain’s susceptibility to clarithromycin, ethambutol, and rifabutin (MICs <4.0, <1.25, and <0.12, respectively); intermediate resistance to rifampin and amikacin (MIC 4.0); and resistance to moxifloxacin and ciprofloxacin (MIC >4.0) and to kanamycin (MIC >8.0). Clinical improvement occurred after 8 weeks of treatment; the condition resolved after 6 months (Figure, panels C, D). Treatment was continued for 12 months.

Five other cases of *M. arupense* tenosynovitis have been reported (*2*,*4*,*5*,*7*,*8*); all patients were immunocompetent or minimally immunocompromised (i.e., diabetes mellitus) (Technical Appendix). The hand was the site of infection in all cases, and 4 of 5 patients reported prior trauma to the affected area, which suggests that inoculation was the infection mechanism. In the case we describe, the disease appeared to progress much faster than in the immunocompetent patients (weeks vs. months to years). Acid-fast bacilli stain was negative in all of the cases where it was performed (*2**,**7**,**8*; this study), and growth on solid Löwenstein-Jensen stain or Middlebrook media was seen after a prolonged incubation time, ranging from 27 days to 2 months. Liquid culture media appears to be unreliable for the growth of *M. arupense* (*8*; this study).

A combination of tenosynovectomy and prolonged antimycobacterial treatment, guided by in vitro strain susceptibility, was used in all the reported cases; a positive outcome was achieved in 6–14 months. The strain susceptibility results we found are comparable with those in the previous cases, showing consistent susceptibility to clarithromycin, ethambutol, and rifabutin; variable susceptibility to linezolid, streptomycin, and amikacin; and resistance to rifampin and quinolones.

Two cases of *M. arupense* infection have been reported in immunosuppressed persons, both in HIV/AIDS patients (manifesting as pulmonary infection in 1 patient and disseminated disease in the other) (*6*). In our study, the immunocompromised patient with *M. arupense* tenosynovitis received canakinumab, a relatively new biologic agent with a prolonged selective IL-1 β-blockade. Even though the contribution of canakinumab in this case is confounded by concomitant immune deficiencies (natural killer cell deficiency, high-dose corticosteroids), the temporal association between initiation of canakinumab and the onset of symptoms raises concern of a possible association. Animal studies have shown that IL-1 plays a key role in host resistance to mycobacterial infections by regulating Th1/Th2 immune responses and inducing granuloma formation (*9*). Clinical trials and systematic reviews assessing the safety of IL-1 inhibitors, including anakinra, rilonacept, and canakinumab, have not shown that these drugs lead to an increased risk of tuberculosis or other mycobacterial infections (*10*). Nonetheless, our report provides increased evidence that *M. arupense* is an emerging cause of tenosynovitis and that it is potentially associated with immunosuppression.

Technical Appendix Clinical characteristics and microbiological and treatment characteristics of case-patients with *Mycobacterium arupense* tenosynovitis in published reports.
