# Supplementary material for: Mycobacterium arupense as an Emerging Cause of Tenosynovitis
Source: Emerg Infect Dis. 2016 Mar;22(3):559–61. doi: 10.3201/eid2203.151479 (PMC4766891; doi:10.3201/eid2203.151479)
Supplement: Technical Appendix — Clinical characteristics and microbiological and treatment characteristics of case-patients with Mycobacterium arupense tenosynovitis in published reports. [file 15-1479-Techapp-s1.pdf]

Article DOI: http://dx.doi.org/10.3201/eid2203.1479

Mycobacterium arupense as an Emerging Cause of Tenosynovitis

Technical Appendix

Technical Appendix Table 1. Patient clinical characteristics of case-patients with *Mycobacterium arupense* tenosynovitis* in published reports

Report	Year	Country	Sex, age, y	Coexisting condition	Presentation	Initial event	Risk for progression	Symptom duration, wk†
Tsai et al. (*1*)	2008	Taiwan	F, 54	Diabetes mellitus	TS/hand	Blunt trauma		28
Senda et al. (*2*)	2011	Japan	M, 68	Hypertension	TS/hand	None	Corticosteroid injection	20
Legout et al. (*3*)	2012	France	M, 35	None	TS and OM/ wrist	Penetrating trauma (glass)	Corticosteroid injection	68
Lee et al. (*4*)	2014	North Korea	F, 56	Hypertension, resected pituitary adenoma. Receiving low-dose prednisolone.	TS/hand	Penetrating trauma (by crab)	Corticosteroid injection	44
Beam et al. (*5*)	2014	USA	M, 58	None	TS/hand	Remote blunt trauma	Systemic corticosteroids, methotraxate, adalimubab	100
This report	2015	USA	M, 62	NK cell deficiency, hyper IL-6 syndrome, recurrent polychondritis, Sweet syndrome. Receiving high- dose prednisone and canakinumab.	TS/hand and wrist	None	Increasing dose of systemic corticosteroids	6

*TS, tenosynovitis; OM, osteomyelitis; NK, natural killer; IL, interleukin.
†Time reported from initial symptoms to diagnosis.

Technical Appendix Table 2. Microbiological and treatment characteristics of case-patients with *Mycobacterium arupense* tenosynovitis in published reports *

Case	Pathology	Culture	Identification method	Susceptibility	Resistance	Treatment	Treatment duration, mo	Outcome
Tsai et al. (*1*)	Granulomas+, AFB–	L-J positive at 60 d	16S rRNA, hsp65 and rpoB	NR	NR	Synovectomy. clarithromycin, ethambutol, rifabutin, moxifloxacin, ciprofloxacin	6	Resolved
Senda et al. (*2*)	Granulomas+, AFB NR	L-J negative	DNA-DNA hybridization	NA	NA	Synovectomy. ethambutol, rifampin	14	Resolved
Legout et al. (*3*)	Granulomas+, AFB NR	L-J positive	16S rRNA and hsp65	NR	NR	Synovectomy and arthrodesis. Clarithromycin, ciprofloxacin, amikacin (1 mo), ethambutol (2 mo)	12	Resolved
Lee et al. (*4*)	Granulomas+, AFB–	L-J positive at 27 d	16S rRNA and hsp65	Clarithromycin, ethambutol, linezolid	Ciprofloxacin, moxifloxacin, rifampin,	Synovectomy. clarithromycin, ethambutol, rifampin	NR	Resolved

Case	Pathology	Culture	Identification method	Susceptibility	Resistance	Treatment	Treatment duration, mo	Outcome
					amikacin, TMP/SMX			
Beam et al. (*5*)	Granulomas–,AFB–	MGIT negative L-J positive at 33 d	16S rRNA	Clarithromycin, ethambutol, rifabutin	Ciprofloxacin, moxifloxacin, rifampin, amikacin, TMP/SMX, linezolid	Synovectomy. clarithromycin, ethambutol, rifabutin	6, ongoing	Improved
This study	Granulomas–,AFB–	MGIT negative L-J positive at 35 d	16S rRNA			Synovectomy. clarithromycin, ethambutol, rifabutin	12	Resolved

*AFB, acid-fast bacilli staining; L-J, Löwenstein-Jensen culture; MGIT, mycobacteria growth indicator tube; NR, not reported; NA, not applicable; TMP/SMX, trimethoprim/sulfamethoxazole.
†Time reported from initial symptoms to diagnosis.

References

1. Tsai TF, Lai CC, Tsai IC, Chang CH, Hsiao CH, Hsueh PR. Tenosynovitis caused by *Mycobacterium arupense* in a patient with diabetes mellitus. Clin Infect Dis. 2008;47:861–3. PubMed http://dx.doi.org/10.1086/591281

2. Senda H, Muro H, Terada S. Flexor tenosynovitis caused by *Mycobacterium arupense*. J Hand Surg Eur Vol. 2011;36:72–3. PubMed http://dx.doi.org/10.1177/1753193410381825

3. Legout L, Ettahar N, Massongo M, Veziris N, Ajana F, Beltrand E, et al. Osteomyelitis of the wrist caused by *Mycobacterium arupense* in an immunocompetent patient: a unique case. Int J Infect Dis. 2012;16:e761–2. PubMed http://dx.doi.org/10.1016/j.ijid.2012.05.007

4. Lee SJ, Hong SK, Park SS, Kim EC. First Korean case of *Mycobacterium arupense* tenosynovitis. Ann Lab Med. 2014;34:321–4. PubMed http://dx.doi.org/10.3343/alm.2014.34.4.321

5. Beam E, Vasoo S, Simner PJ, Rizzo M, Mason EL, Walker RC, et al. *Mycobacterium arupense* flexor tenosynovitis: case report and review of antimicrobial susceptibility profiles for 40 clinical isolates. J Clin Microbiol. 2014;52:2706–8. PubMed http://dx.doi.org/10.1128/JCM.00277-14
